# Three-dimensional analysis of mandible ramus morphology and transverse stability after intraoral vertical ramus osteotomy

**DOI:** 10.1007/s00276-022-02912-z

**Published:** 2022-03-18

**Authors:** Luo Huang, Shan Tang, Jing Yan, Yaoran Liu, Zhengguo Piao

**Affiliations:** 1 Guangzhou Key Laboratory of Basic and Applied Research of Oral Regenerative Medicine, Key Laboratory of Oral Medicine, Department of Oral and Maxillofacial Surgery, Guangzhou Institute of Oral Disease, Affiliated Stomatology Hospital of Guangzhou Medical University, No. 39 Huangsha Avenue, Guangzhou, 510150 China; 2 Stomatology Department, Zhujiang Hospital of Southern Medical University, Guangzhou, 510282 China

**Keywords:** Intraoral vertical ramus osteotomy (IVRO), Mandibular ramus, Bone morphology, Transverse stability

## Abstract

**Objectives:**

The purpose of this study was to investigate short- and long-term postoperative changes of both morphology and transverse stability in mandibular ramus after intraoral vertical ramus osteotomy (IVRO) in patients with jaw deformity using three-dimensional (3D) orthognathic surgery planning treatment software for measurement of distances and angles.

**Study design:**

This retrospective study included consecutive patients with skeletal Class III malocclusion who had undergone intraoral vertical ramus osteotomy and computed tomography images before (T0), immediately after (T1), and 1 year after (T2) surgery. Reference points, reference lines and evaluation items were designated on the reconstructed 3D surface models to measure distances, angles and volume. The average values at T0, T1, T2 and time-dependent changes in variables were obtained.

**Results:**

After surgery, the condylar length, ramal height, mandibular body length and mandibular ramus volume were significantly decreased (*P* < 0.01), while clinically insignificant change was observed from T1 to T2. The angular length was increased immediately after surgery (*P* < 0.05), but it was decreased 1 year after surgery (*P* < 0.05). Lateral ramal inclination showed significant increase after surgery (*P* < 0.05) and maintained at T2.

**Conclusion:**

Changes in the morphology of the mandibular ramus caused by IVRO do not obviously bring negative effect on facial appearance. Furthermore, despite position and angle of mandibular ramus changed after IVRO, good transverse stability was observed postoperatively. Therefore, IVRO technique can be safely used without compromising esthetic results.

## Introduction

As patients have begun to express more interest in the esthetic results of their surgeries, surgeons must be able to reliably predict any differences in appearance that may result from orthognathic surgeries. To achieve better outcomes, surgery must be performed with consideration of the extent of mandibular morphology changes that may occur postoperatively. It is extremely important to fully understand the complex structural anatomy of the mandible, including the external surface, the position and thickness of the mandibular foramen, the thinning of the mandibular ramus buccolingually, and the course of the mandibular canal, when performing orthognathic surgery. Moreover, some anatomical variations of the mandible cannot be neglected, such as the accessory mandibular foramen [8].These knowledge will help the surgeon adopt the necessary surgical procedures to reduce the incidence of adverse events and also improve the position and length of the mandible both esthetically and functionally. However, full knowledge of mandibular anatomy notwithstanding, the surgical procedure for mandibular setback positions proximal segments laterally to distal segments, which may influence esthetic appearance after orthognathic surgery [4].

Intraoral vertical ramus osteotomy (IVRO) is a common surgical procedure for treating prognathic mandibles, and it offers several advantages. In IVRO, the ramus is cut behind the mandibular foramen from the sigmoid notch to the mandibular angle, and the external appearance of both the ramus and mandibular angle is changed. Because the split segments are not fixed and the contact area between them is small, some clinicians have raised concerns that IVRO would increase the transverse mandibular width because the proximal segment is lateralized to the distal segment [9]. Moreover, protrusion of the proximal segment can cause the mandibular angle to protrude toward the buccal side and change the gonial angle because the vertical osteotomy line is more posterior in IVRO [13, 14]. Thus, IVRO may lead to unfavorable esthetic results. Many studies have examined temporal changes in the morphology of the mandibular ramus after IVRO [1, 9]. To the best of our knowledge, most of these studies were reported using a two-dimensional imaging analysis, which made it difficult to arrive at an accurate evaluation due to jaw bone overlap in the profile. Hence, the present study aimed to measure the dimensional changes of the mandibular ramus as well as the transverse stability of the mandibular ramus after IVRO in patients with jaw deformity using a three-dimensional (3D) computed tomography (CT) image processing system for the first time.

## Materials and methods

### Participants

This study involved patients who were treated with mandibular setback surgery using IVRO at the Department of Oral and Maxillofacial Surgery of the Affiliated Stomatology Hospital of Guangzhou Medical University from July 2015 to August 2018. Due to the retrospective nature of this study, it was granted an exemption in writing by the Affiliated Stomatology Hospital of Guangzhou Medical University IRB. Only patients who underwent facial 3D CBCT at the preoperative, 1-week postoperative, and 1-year postoperative stages were included in this study. A total of 27 patients (11 males and 16 females; mean age, 20.68 years; range 18–33 years) were selected. All patients were free of congenital diseases and syndromes. Patients with a history of orthognathic surgery, cleft lip or palate, craniofacial syndromes, history of trauma, or facial asymmetry (where the midline of menton showed left–right deviation by 3.5 mm or more [6]) were excluded.

### Surgical procedure

**Fig. 1:**
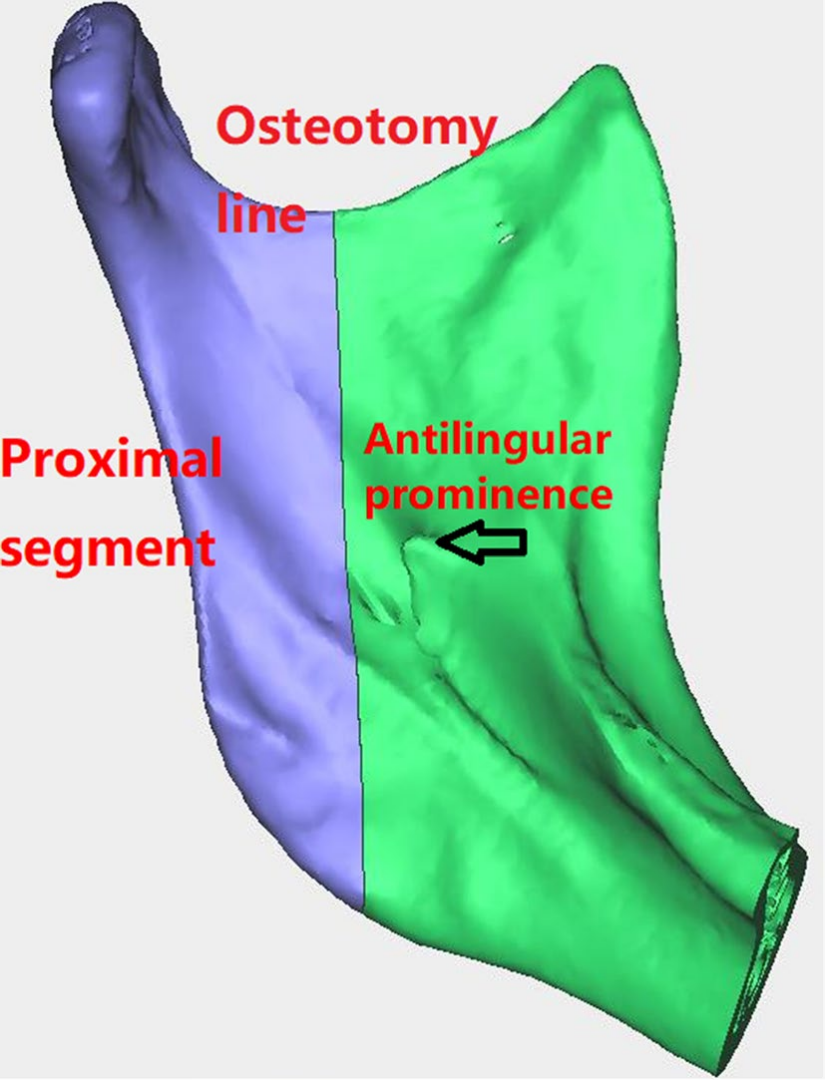
The illustration of surgical technique of intraoral vertical ramus osteotomy (IVRO)

The initial incision was made from the anterior border of the ascending ramus to the external oblique line at the level of the second molar. A Bauer retractor was placed to protect the contents and to antilingular prominence. A trial osteotomy was marked 4–5 mm posterior to the antilingular prominence. A vertical osteotomy with a full thickness cut is made with a sagittal saw from the midsigmoid notch area inferior to the antilingular prominence and then was directed anteriorly to maximize proximal segment width. The osteotomy was completed through the inferior border of the mandible, and separation of the proximal segment and distal segment was confirmed. Using a round bur, the medial cortical edge of the proximal segment was trimmed to achieve the planned setback and segment overlap (Fig. 1).

### Data acquisition

**Fig. 2:**
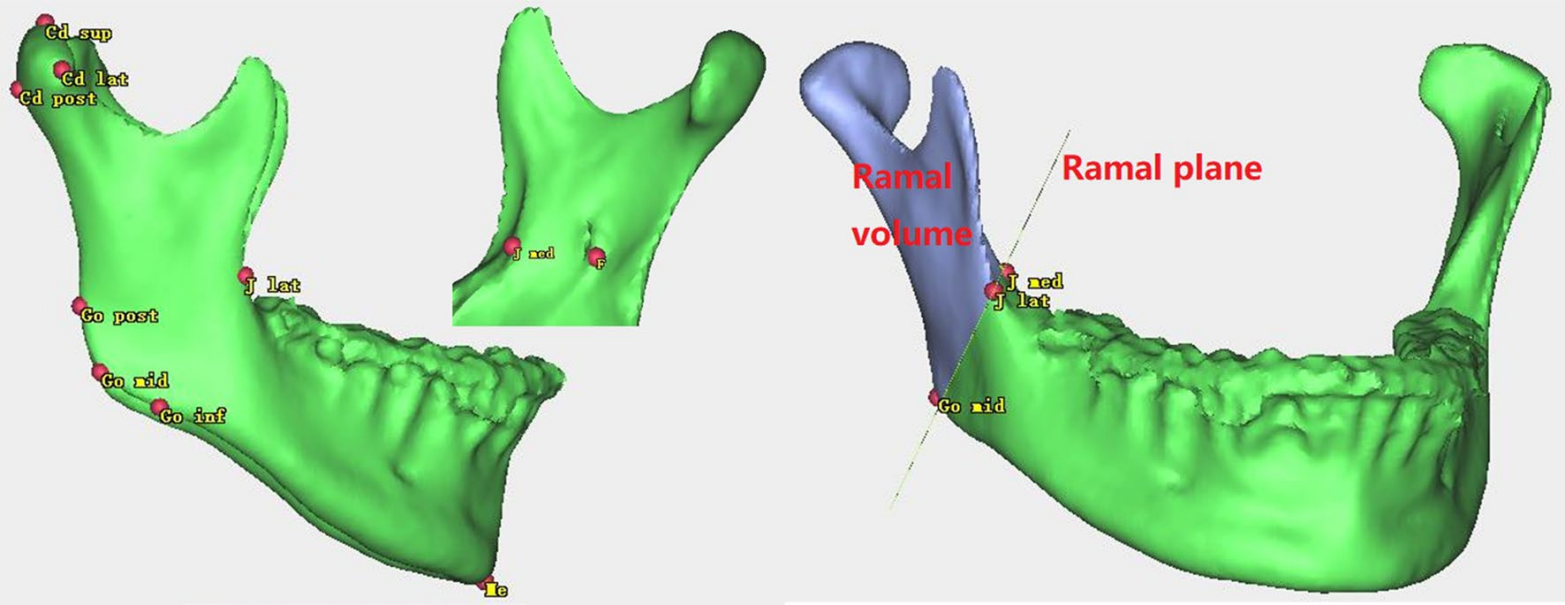
Reference points for 3D analysis in this study. *Cd*_*sup*_ Condylion_superior, *Cd*_*post*_ Condylion_posterior, *Me* Menton, *F* Fossa of mandibular foramen, *Go*_*post*_ Gonion posterius, *Go*_*mid*_ Gonion midpoint, *Go*_*inf*_ Gonion inferius, *J*_*lat*_ Lateral and deepest point of the curvature, *J*_*med*_ media and deepest point of the curvature

3D facial CT images were obtained with a NewTom scanner (Imaging Science International, Hatfield, PA, Italy) using a 200–400 mm field of view, 120 kVp, and 47.7 mA, resulting in a 0.4-mm voxel size; preoperative, 1-week postoperative, and 1-year postoperative stages were designated as T0, T1, and T2, respectively. ProPlan CMF 3.0 (Materialize, Leuven, Belgium) was used to conduct 3D image reconstruction and 3D analysis of the images. After reconstruction of the 3D skull model, the Frankfort horizontal plane (FH) was used as the horizontal reference plane passing through the bilateral orbitales and right porion. The midsagittal plane was defined as the midline reference plane intersecting the nasion and sella and was perpendicular to the FH. Landmarks in this study (Fig. 2) were designated on the reconstructed 3D surface model and are detailed in Table 1, and these were selected in accordance with the procedure used in a previous study [3, 15]. Reference planes and measurements are presented in Tables 2 and 3, respectively.

**Table 1:** Landmarks used in this study

Landmarks	Description
N (Nasion)	The midpoint of the frontonasal structure—the intersection of the internasal and frontonasal sutures in the midsagittal plane
Se (Sella turcica)	The center of the hypophyseal fossa
Po (Porion)	Highest midpoint of the roof of the external auditory meatus
Or (Orbitale)	Lowest point of the infraorbital margin of the orbit
Cd_sup_ (Condylion_superior)	Most superior point of the condyle head
Cd_lat_ (Condylion_lateral)	Most lateral point of the condyle head
Cd_post_ (Condylion_posterior)	Most posterior point of the condyle head
Me (Menton)	Most inferior point of the symphyseal outline
F (Fossa of mandibular foramen)	The most inferior point on the fossa of the mandibular foramen
J_lat_	The most lateral and deepest point of the curvature formed at the junction of the mandibular ramus and body
J_med_	The most medial and deepest point of the curvature formed at the junction of the mandibular ramus and body
Go_post_ (Gonion posterius)	The most posterior point on the mandibular angle
Go_mid_ (Gonion midpoint)	The midpoint between Go_post_ and Go_inf_ on the mandibular angle
Go_inf_ (Gonion inferius)	The most inferior point on the mandibular angle

**Table 2:** Reference planes used in this study

Planes	Description
FH plane (Frankfort horizontal plane)	The plane passing through both sides of the Or and right side of Po
MSP plane (Midsagittal reference plane)	The Plane through Se and N and perpendicular to FH
Ramal plane	The plane passing through Go_mid_, J_lat_, and J_med_

**Table 3:** Measurements used in this study

Measurements	Definition
Condylar unit length (mm)	Distance between Con_sup_ and F
Angular unit length (mm)	Distance between F and Go_mid_
Ramal height (mm)	Distance between Con_sup_ and Go_mid_
Mandibular body length (mm)	Distance between Go_mid_ and Me
Frontal ramal inclination (°)	Angle formed by Cd_lat_–Go_mid_ to MSP plane
Lateral ramal inclination (°)	Angle formed by Cd_post_–Go_post_ to FH plane
Ramal volume diff (3 × 10^3^ mm^3^)	Volume of mandibular ramus

### Statistical analysis

To avoid investigator-related bias, 10 CT images were randomly selected and measured twice by one investigator in a 10-day interval using intra-class correlation. SPSS software for Windows (version 21.0; IBM Corp., Armonk, NY, USA) was used for statistical analyses. The average value and standard deviation for each measurement were calculated to detect the amount of change over time, and changes between T0 and T1, between T1 and T2, and between T2 and T1 were calculated using paired *t* tests. Differences were considered significant at *P* < 0.05.

## Results

### Pre-surgery

**Fig. 3:**
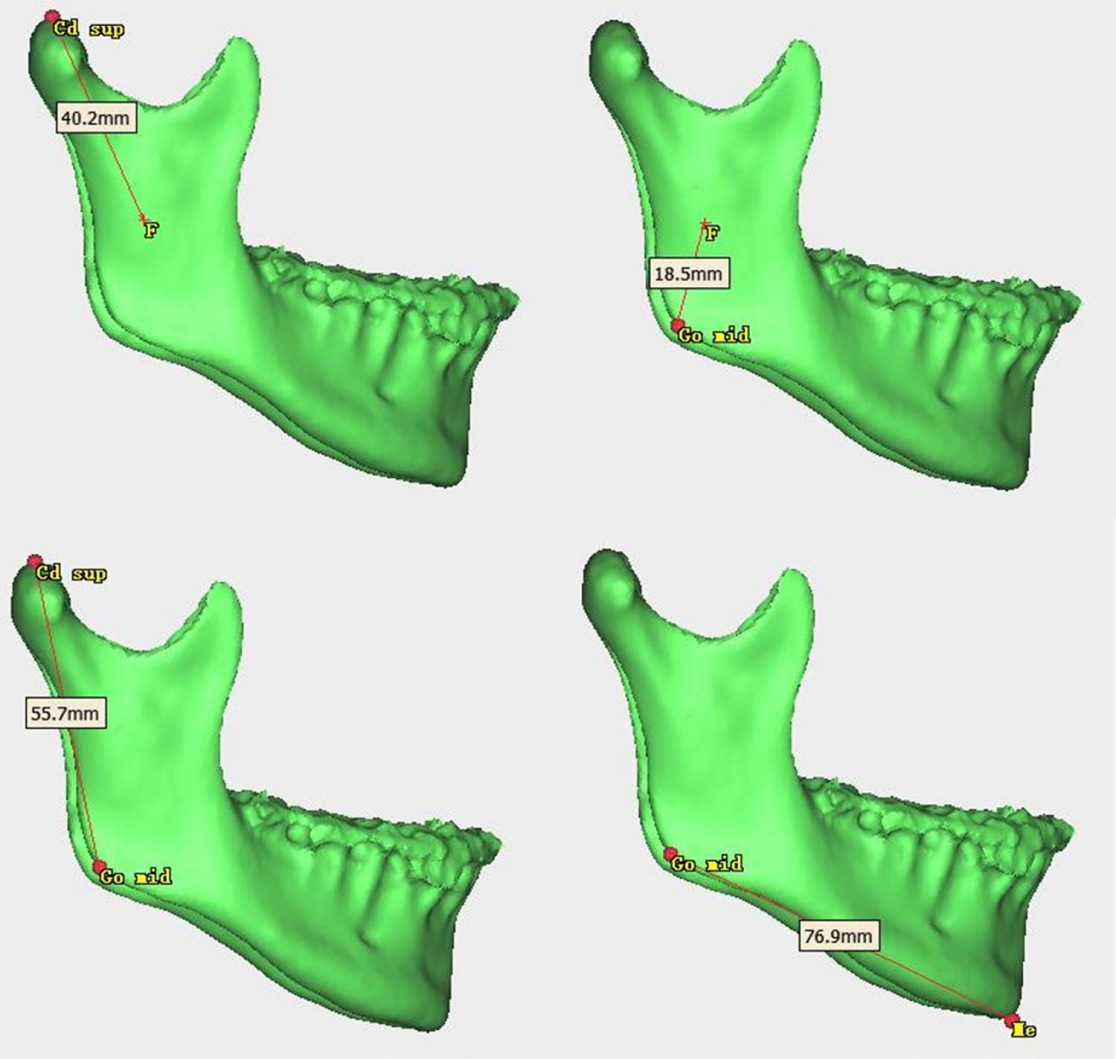
Measurements distance for 3D analysis in this study. **A** Condylar unit length; **B** angular unit length; **C** ramal height; **D** mandibular body length

**Fig. 4:**
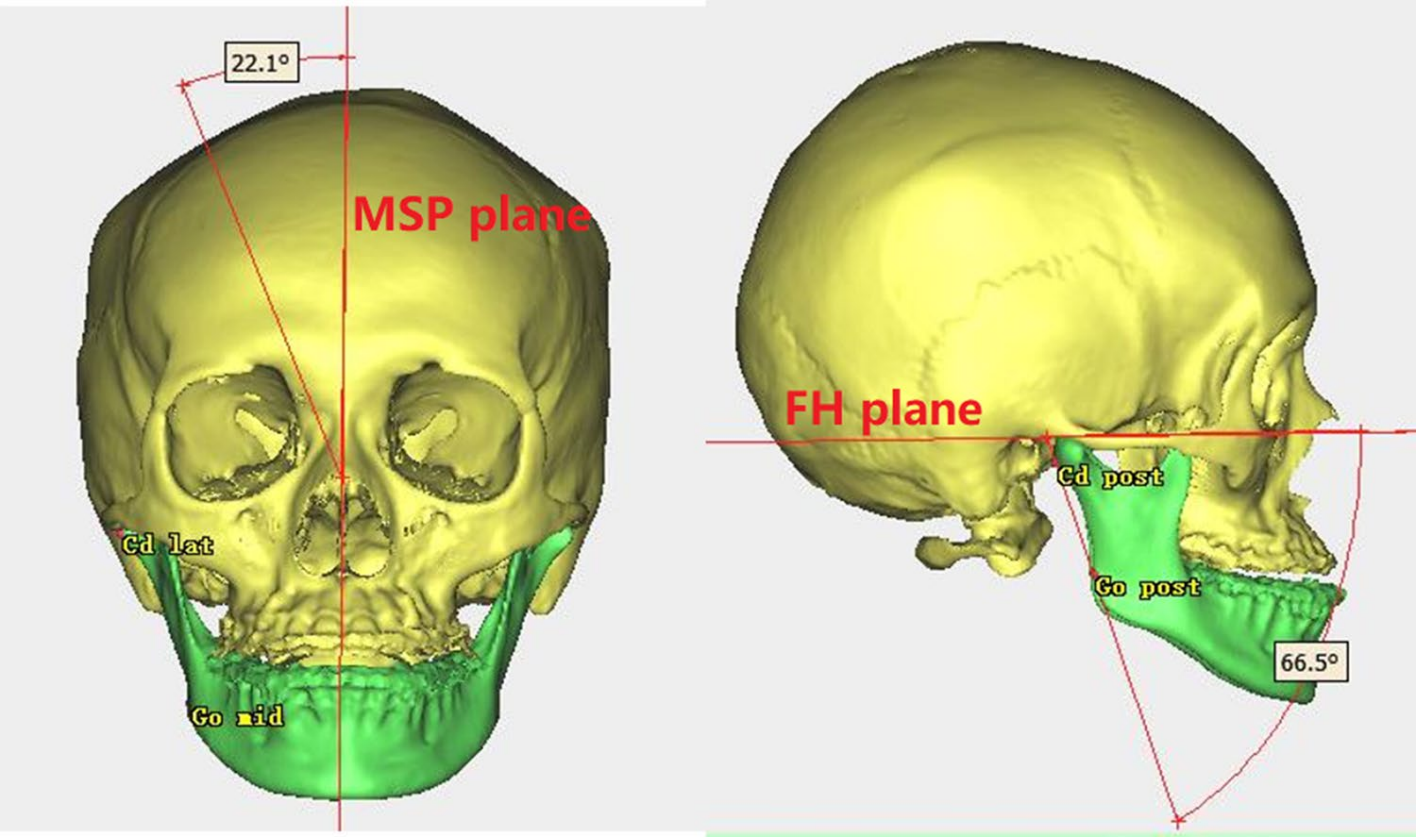
Measurements angle for 3D analysis in this study. **A** Frontal ramal inclination; **B** lateral ramal inclination

Among the four distance measurements, the average value and standard deviation for condylar length (Fig. 3A), angular length (Fig. 3B), and ramal height (Fig. 3C) were not obviously different between the right and left sides before surgery. Pre-surgery mean values of the mandibular body length (Fig. 3D) were 83.60 ± 6.66 mm for the right side and 91.90 ± 5.78 mm for the left side, indicating pre-existing mandibular body length discrepancies. With respect to mandibular ramus volume (Fig. 2B), the left side with a longer mandibular body length was smaller than the other side, which probably reflected mandibular ramus morphology compensation for the mandibular body. Frontal ramal inclination (Fig. 4A) also showed a tendency similar to the mandibular ramus volume. For lateral ramal inclination (Fig. 4B), the mean values of both sides exhibited essentially the same condition (Table 4).

**Table 4:** 3D analysis to evaluate the skeletal stability after surgery

Measurements	T0	T1	T2
Condylar unit length (R) (mm)	45.20 ± 3.93	36.60 ± 3.93	35.30 ± 3.69
Condylar unit length (L) (mm)	44.40 ± 4.01	36.90 ± 3.83	36.60 ± 4.33
Angular unit length (R) (mm)	19.60 ± 2.95	20.50 ± 3.75	20.00 ± 3.31
Angular unit length (L) (mm)	19.40 ± 2.50	20.70 ± 4.17	20.30 ± 3.75
Ramal height (R) (mm)	59.80 ± 5.45	53.40 ± 12.76	53.20 ± 4.94
Ramal height (L) (mm)	58.00 ± 5.27	51.80 ± 4.60	52.30 ± 5.45
Mandibular body length (R) (mm)	87.60 ± 6.66	83.50 ± 8.29	82.20 ± 7.17
Mandibular body length (L) (mm)	91.90 ± 5.78	88.30 ± 6.47	86.60 ± 5.40
Ramal volume diff (R) (mm^3^)	9250 ± 2367	6254 ± 1781	6548 ± 2090
Ramal volume diff (L) (mm^3^) (°)	8530 ± 2409	6350 ± 1803	6667 ± 2112
Frontal ramal inclination (R) (°)	15.80 ± 4.36	16.40 ± 5.13	17.10 ± 5.34
Frontal ramal inclination (L) (°)	14.60 ± 4.62	15.50 ± 4.29	17.90 ± 5.34
Lateral ramal inclination (R) (°)	77.00 ± 3.92	79.60 ± 15.41	77.40 ± 17.46
Lateral ramal inclination (L) (°)	76.80 ± 4.10	80.80 ± 3.97	78.20 ± 6.12

### Surgical and postoperative changes

After surgery, the condylar and ramal heights were decreased by superior positioning of the distal segment (*P* < 0.01). The mandibular body lengths significantly decreased because of the mandibular setback on both sides from T0 to T1 (*P* < 0.05), while clinically insignificant changes were observed from T1 to T2. The angular length increased immediately after surgery (*P* < 0.05) but decreased 1 year after surgery (*P* < 0.05). Most distance measurements did not show significant differences from T2 to T1, except for the angular length. The mandibular ramus volume was significantly reduced on both sides (*P* < 0.01) and was maintained 1 year after surgery. Lateral ramal inclination was significantly increased after surgery (*P* < 0.05), with the mandibular ramus inclined forward considerably. Compared to T0, lateral ramal inclination also increased at T2, although this was not statistically significant. Frontal ramal inclination on both sides showed no significant changes during the postoperative period (Table 5).

**Table 5:** Analysis for surgical and postoperative changes

Measurements	Surgical changes		Postoperative changes		Total change	
	T1–T0	*P*	T2–T1	*P*	T2–T0	*P*
Condylar unit length (R) (mm)	– 9.64 ± 3.76	< 0.01	– 1.14 ± 3.61	0.21	– 10.77 ± 4.65	< 0.01
Condylar unit length (L) (mm)	– 7.38 ± 4.07	< 0.01	– 0.33 ± 2.60	0.60	– 7.71 ± 3.89	< 0.01
Angular unit length (R) (mm)	1.82 ± 3.46	< 0.05	– 1.816 ± 3.46	< 0.05	0.012 ± 2.12	0.98
Angular unit length (L) (mm)	2.27 ± 3.58	< 0.05	2.27 ± 3.46	< 0.01	– 0.74 ± 2.34	0.21
Ramal height (R) (mm)	– 8.88 ± 12.38	< 0.01	0.27 ± 11.56	0.93	– 8.61 ± 4.80	< 0.01
Ramal height (L) (mm)	– 5.00 ± 4.59	< 0.01	– 2.76 ± 3.98	0.05	– 7.76 ± 3.925	< 0.01
Mandibular body length (R) (mm)	2.73 ± 5.42	< 0.05	– 2.89 ± 6.63	0.09	– 0.15 ± 6.913	< 0.05
Mandibular body length (L) (mm)	– 2.61 ± 4.04	< 0.05	– 1.36 ± 3.58	0.15	– 3.986 ± 3.24	< 0.01
Ramal volume diff (R) (mm^3^)	– 1830 ± 2239	< 0.01	8532 ± 2367	0.62	– 1690 ± 1864	< 0.01
Ramal volume diff (L) (mm^3^)	– 1864 ± 2341	< 0.01	305.0 ± 1431	0.39	– 1559 ± 1638	< 0.01
Frontal ramal inclination (R) (°)	0.64 ± 2.89	0.38	0.57 ± 3.29	0.49	1.21 ± 3.38	0.16
Frontal ramal inclination (L) (°)	1.33 ± 2.49	0.09	1.49 ± 3.30	0.08	2.82 ± 2.78	0.07
Lateral ramal inclination (R) (°)	– 0.38 ± 16.08	< 0.05	– 2.23 ± 25.11	0.72	– 2.61 ± 16.60	0.53
Lateral ramal inclination (L) (°)	4.95 ± 5.52	< 0.01	– 3.54 ± 6.95	0.05	1.41 ± 3.39	0.11

## Discussion

IVRO is a widely known surgical technique for correcting mandibular prognathism. However, the IVRO technique for mandibular setback has a potential esthetic disadvantage because the two intact segments overlap and their thickness doubles, resulting in a changed frontal appearance. The present study revealed morphological changes in the mandibular ramus in IVRO. The characteristics of bone overlap are apparent as the ramus height is significantly decreased and the external appearance of the mandible changes considerably. Therefore, the changes in external appearance associated with the changes in ramus produced by IVRO are obvious. However, ramus height did not show a significant change 1 year after surgery, resulting in patients having a smoother profile. This is consistent with a report that found that frontal mandibular width increased after IVRO but seemed to normalize within approximately 3 years [2]. Post-IVRO protrusion of the mandibular angle due to protrusion of the proximal segment is considered a major problem associated with IVRO. Jung et al. examined changes in mandibular width after IVRO [9]. They observed significant differences in cephalometric radiographs during the first postoperative year, suggesting that mandibular angle protrusion after IVRO should not be considered a problem. However, they used cephalometric radiographs to evaluate bone changes, which is thought to make it difficult to accurately measure the position due to overlap. In the present study, CT images were used to measure the changes in the length of the mandibular angles before and after IVRO. The results showed that the mandibular angle length was approximately the same 1 year postoperatively as before the surgery. This indicates that while a slight increase may persist, there is no need to consider how the face may be affected by mandibular angle protrusion due to protrusion of the proximal segments. The decrease in ramal volume at the 1-year postoperative stage compared with the 1-week postoperative stage in this study could be the result of bone remodeling after periosteal stripping at the medial side of the proximal segment in the gonial region [11].

Stability is one of the most important criteria for determining treatment success in orthognathic surgery, and clinicians and patients expect effective long-term results. Previous studies have shown that the IVRO technique causes skeletal antero-inferior condylar displacement [4] and proximal segment posterior drift [5], resulting in unpredictability of the postoperative mandibular position. Pan et al. [11] demonstrated that the ramus angle and ramus inclination angle increased after IVRO and then regressed. The present study showed that lateral ramal inclination was larger postoperatively, indicating posterior displacement at the tip of the proximal segment. This displacement of the proximal segment may affect skeletal stability. However, IVRO does not fixate the proximal and distal segments, so the lateral ramal inclination returns to the new physiological position based on its functional needs and the action of bone remodeling. This position corresponds to bone movement and lateral pterygoid muscle attachment; thus, the postoperative lateral ramal inclination does not change significantly [10, 12]. Despite the significant decrease in ramal height after IVRO in our study, the condylar length and angular length exhibited a complementary relationship; hence, the small change in the lateral ramal inclination is reasonable.

Patients with mandibular prognathism present with anterior crossbite, which leads to difficulty with mastication. A study showed that the height of the mandibular ramus decreased significantly in individuals with a hyperbranched skeletal pattern [7].Treatment for patients with mandibular prognathism requires not only mandibular setback to correct the malocclusion and restore the masticatory function but also consideration of the harmony of facial patterns after surgery. Symmetricity in frontal ramal inclination is important, especially in patients with facial asymmetry. In addition, as postoperative changes in the frontal ramal inclination may lead to reappearance, it is necessary to maintain improved frontal ramal inclination postoperatively.

## Conclusion

In conclusion, changes in the morphology of the mandibular ramus caused by IVRO do not have a negative effect on facial appearance. This study included symmetric patients who underwent mandibular setback surgery via IVRO and then focused on postoperative changes and maintenance. Even though frontal ramal inclination slightly increased after IVRO, there was no significant difference during the postoperative period. This indicated that lateral flare and remodeling of the proximal segment after IVRO might have decreased the difference in frontal ramal inclination [11]. However, as the distal segment cannot be corrected by IVRO, the preoperative difference would be maintained in the frontal ramal inclination, even 1 year after surgery. Furthermore, despite changes in the position and angle of the mandibular ramus after IVRO, good transverse stability was observed postoperatively. Therefore, IVRO technique can be safely used without compromising esthetic results.

Our data suggest that there are no significant esthetic changes in patients after IVRO and that using 3D imaging is a promising way to measure any such changes. Previous research used cephalometric radiographs, which presented challenges due to the nature in which jaw bones overlapped in the profile. This study was able to make more accurate measurements using 3D imaging (CT scans). Further research should be conducted using 3D imaging to measure surgical results at additional hospitals and under varying conditions.

## Data Availability

The data sets supporting the results of this article are included within the article and its additional files.
